# Effect of antenatal breast milk expression or postnatal dietary intervention on breastfeeding outcomes and human milk composition in women with excess weight: protocol for the Ready, Set, Nourish study, a randomized controlled trial

**DOI:** 10.3389/fnut.2026.1854997

**Published:** 2026-09-18

**Authors:** Larissa Leandro da Cruz, Chelsey Fiecke, Anni Fuenmayor, Emily Beckett, Andrew Morris, Colin D. Kay, Taren Massey-Swindle, Sarah Sobik, Aline Andres

**Affiliations:** 1 Arkansas Children’s Nutrition Center, Little Rock, AR, United States; 2 Department of Pediatrics, University of Arkansas for Medical Sciences, Little Rock, AR, United States; 3 Arkansas Children’s Hospital, Southwest Little Rock Community Clinic, Little Rock, AR, United States; 4 Department of Pharmacology and Toxicology, University of Arkansas for Medical Sciences, Little Rock, AR, United States

**Keywords:** antenatal breastmilk expression, breastfeeding, colostrum, dietary intervention, human milk, lactation support, obesity, pregnancy

## Abstract

**Background:**

Human milk is recommended as the ideal source of nutrients intake for infants. It provides optimal nutrition and critical health benefits for both infants and mothers. However, women with excess weight are less likely to initiate and sustain breastfeeding. This study aims to assess how a prenatal and a postnatal intervention can improve breastfeeding outcomes, human milk composition, and infant growth in women with excess weight.

**Methods:**

This three-arm randomized controlled trial will include 180 pregnant women with pre-pregnancy BMI ≥ 25 kg/m^2^ recruited from central Arkansas. The participants will be randomized to a standard of care group receiving standardized lactation support (*N* = 60); a prenatal intervention group receiving standardized lactation support + antenatal breast milk expression (ABME) between 37 weeks of gestation until birth (*N* = 60); or a postnatal intervention group receiving standardized lactation support + dietary meal plan designed to align with the Dietary Guidelines for Americans from 2 weeks to 6 months postpartum (*N* = 60). Study assessments will include repeated measures of maternal and infant anthropometrics, body composition, dietary intake, and archiving of biospecimens (blood, human milk, stool, and urine) during infancy and until 6 months postpartum. The primary outcomes are breastfeeding outcomes (initiation, duration, exclusivity) and human milk composition (macronutrients, energy, and bioactive components). Questionnaires will assess maternal demographics and socioeconomic status, breastfeeding self-efficacy, maternal depression, stress, and quality of life as well as infant feeding intention, and medical lactation history.

**Discussion:**

This randomized control trial will generate novel evidence on the safety, feasibility, and effectiveness of ABME and provision of a healthy postnatal meal plan in a population at elevated risk for breastfeeding challenges. By evaluating clinical and nutritional strategies, this study will advance the understanding of how maternal behavior and physiology influence breastfeeding outcomes, human milk composition and early life programming of infant growth and development. Findings will also address critical gaps identified by the Dietary Guidelines Advisory Committee by guiding nutritional recommendations for women with excess weight. The results will inform future strategies targeting short and long-term maternal-infant health outcomes.

**Clinical trial registration:**

https://clinicaltrials.gov/study/NCT00616395, identifier NCT00616395.

## 1 Introduction

The American Academy of Pediatrics recommends exclusive breastfeeding for at least 6 months followed by continued breastfeeding with the introduction of complementary foods as long as mutually desired (1). Breastfeeding can play a significant role in improving maternal and child outcomes (2–5), especially in preventing childhood obesity (6). Early initiation of breastfeeding within the first hour after birth can improve breastfeeding outcomes including exclusivity, milk supply, and duration (7). In Arkansas, 74% of infants are breastfed at some point, but only 24% are exclusively breastfed through 6 months. Current research demonstrates lower breastfeeding initiation (8) and shortened duration of breastfeeding in women with obesity (2, 3), which impacts 40% of women of childbearing age in Arkansas (9). Excess weight may also affect milk composition and contribute to early-life programming of long-term child health (10). There are multiple barriers that contribute to women with overweight and obesity meeting their breastfeeding goals (11, 12), including an increased risk of delayed lactogenesis II (13, 14), which is critical for establishing milk supply and predictive of long-term lactation outcomes (14).

Because of a lack of evidenced-based literature for women with excess weight who are interested in breastfeeding (15), the most effective strategies to support women with excess weight in meeting their breastfeeding goals are still unclear (16). Recent research suggests that women with overweight and obesity who receive additional support and education to breastfeed from a physician or health care professional increase their likelihood to initiate and sustain breastfeeding (11), and to exclusively breastfeed at 4–6 weeks (17). Therefore, educational interventions and support from health professionals tailored toward women with overweight and obesity may improve breastfeeding initiation, exclusivity, and duration (11, 12).

Antenatal breastmilk expression (ABME) refers to manual extraction of colostrum in the later weeks of pregnancy (∼37 weeks until birth). ABME has been explored mostly in mothers with gestational diabetes and/or for reduction of newborn hypoglycemia (18, 19). Starting at 37 weeks of gestation, this practice has the potential to hasten the onset of lactogenesis II (18), improve postpartum engorgement, decrease the need for supplementation in infants, and improve exclusive breastfeeding duration up to 6 months postpartum in mothers with diabetes (20). In the DAME trial (*N* = 635) (21), women with diabetes randomized to the ABME intervention, demonstrated no increase in adverse perinatal outcomes associated with ABME and a higher likelihood of infants receiving human milk at their first feed. Similarly, in the EXPRESS-MOM study (*N* = 28) (22), ABME practiced after 34 weeks of pregnancy in healthy women was associated with an increased proportion of infants fed mother’s own milk in the first 24 h post-partum. A recent systematic review identified limited evidence and few research studies that have tested the feasibility of ABME in pregnant women with various risk factors for breastfeeding outcomes (20). Thus, more research is needed regarding the efficacy, feasibility, and acceptability of ABME to help women with excess weight meet their breastfeeding goals. While the safety of ABME has been questioned (23), different studies (21, 22, 24–29) report no increase in maternal or neonatal adverse events associated with ABME when performed at terms (27, 28). These findings are supported by a systematic review and meta-analysis of 10 randomized controlled trials (*n* = 1,672), which reported no significant differences in NICU admissions, gestational age at birth, birthweight, or cesarean delivery rates between ABME and the control groups (26). Adverse events, which varied according to individual experience, included discomfort, pain, embarrassment, difficulty with the expression, and concern with the volume of colostrum, while overall evidence of safety (29, 30).

Dietary intake is another modifiable factor that can impact breastfeeding outcomes and human milk composition (31). Epidemiological and experimental studies support the role of maternal diet in modifying human milk composition via several pathways that modulate milk components directly and indirectly (32–35). Dietary habits, excess weight, and other factors have been associated with compositional changes in human milk, including increased human milk concentrations of leptin, insulin, C-reactive protein, fat, protein, and some oligosaccharides (36–38). Despite such evidence, no specific dietary recommendations currently exist for lactating women with excess weight to promote optimal breastfeeding outcomes, human milk composition, infant growth, and infant body composition. In fact, the report of the Dietary Guidelines for Americans Advisory Committee pointed out numerous gaps in knowledge that limited their ability to provide strong dietary recommendations for pregnant and lactating women with excess weight (39). Prior dietary intervention studies have demonstrated that maternal diet impacts oligosaccharides (HMOs) (40), phytochemicals (41–44), microbiota (45), and hormone (40) composition of human milk. However, most of these studies involved single dietary components, short duration (≤4 weeks), and small sample sizes (<20). Previous intervention studies of a Mediterranean diet and/or adherence to dietary guidelines are sparse, with only a few within-subject intervention studies suggesting that healthy dietary patterns during lactation can lead to changes in concentrations of lactoferrin (46), amino acids (47), HMOs (40), leptin (40), and phytochemicals (41) in human milk. To date, no randomized controlled dietary interventions have tested the impact of adherence to the Dietary Guidelines for Americans (DGA) on breastfeeding outcomes, human milk composition and developmental outcomes. Considering that several factors, including gestational weight gain, breastfeeding duration, timing of complementary feeding, and protein intake in infancy occurring in the first 1,000 days of life are modifiable and represent risk for the development of later childhood obesity (48–50), investigating maternal behavior during the perinatal period represents a critical opportunity for optimizing maternal and infant health outcomes.

The Ready, Set, Nourish study will evaluate the impact of prenatal and postnatal strategies on optimization of maternal health outcomes and infant development in women with overweight and obesity. The impact of the ABME technique on breastfeeding outcomes (initiation and duration) will be evaluated. In addition, this study will evaluate the impact of a postpartum dietary intervention based on the DGA on breastfeeding outcomes and human milk composition. In both interventions as well as the standard of care group, maternal and infant anthropometric and metabolic outcomes will be examined for the first 6 months of life. All the study groups (ABME, postnatal intervention, and standard of care) will receive early and continuous lactation support to standardize this potential confounder factor. Thus, the objectives of the study are to evaluate whether health care professional education about ABME during pregnancy or healthy postnatal dietary patterns can improve breastfeeding outcomes in women with overweight or obesity.

## 2 Methods and analysis

The Ready, Set, Nourish study is a randomized controlled clinical trial (NCT06929091) enrolling women with overweight or obesity to test whether prenatal or postnatal intervention strategies can impact breastfeeding outcomes, human milk composition, and infant growth. This article follows the SPIRIT (Standard Protocol Items: Recommendations for International Trials) guidelines for reporting clinical trial protocols (51). Figure 1 and Table 1 detail the schedule of enrollment, interventions, and assessments.

**FIGURE 1:**
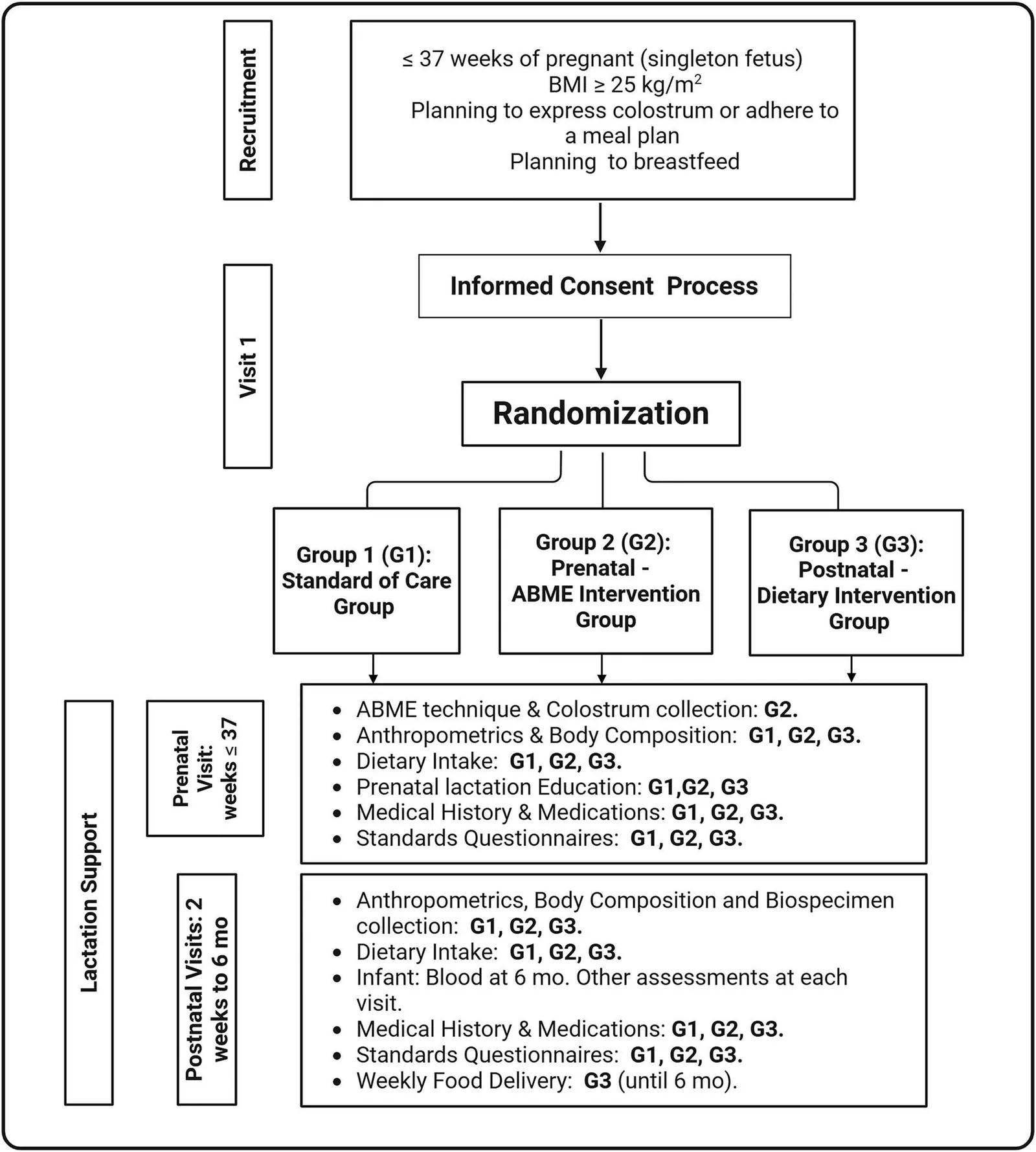
Ready, Set, Nourish study flow chart. Protocol schematic diagram including informed consent, randomization, study groups, and visit assessments.

**TABLE 1:** Schedule of enrollment, interventions, and assessments at specific time points.

		Study period								
		Pregnancy (weeks)		Postpartum (months)						
		Pre-study screening	Pre-study and screening	Post-allocation						
Time point		≤36	37	0.5	1	2	3	4	5	6
Recruitment/enrollment	Team member		Participants							
Eligibility screen	Research team member	M								
Informed consent	Research team member		M							
Allocation of treatment groups	Clinical coordinator or PI		M							
Compensation	Research team member		M	M	M	M	M	M	M	M
**Intervention**	**Team member**		**Participants**							
Prenatal intervention – antenatal breast milk expression	Research team member		M							
Postnatal intervention – diet intervention	Research team member			M	M	M	M	M	M	M
Standard of care	Research team member		M	M	M	M	M	M	M	M
**Assessments**	**Team member**		**Participants**							
Demographic and contact information	Research team member		M							
Medical history	Research team member		M	M/I	M/I	M/I	M/I	M/I	M/I	M/I
Vital signs	Research team member		M	M	M	M	M	M	M	M
Anthropometrics and body composition	Research team member		M	M/I	M/I	M/I	M/I	M/I	M/I	M/I
Fasted blood collection	Phlebotomist			M	M	M	M	M	M	M/I
Urine sample collection	Participant			M/I	M/I	M/I	M/I	M/I	M/I	M/I
Stool sample collection	Participant		M	M/I	M/I	M/I	M/I	M/I	M/I	M/I
Human milk sample collection	Participant			M	M	M	M	M	M	M
Toenail collection	Participant						M			M
Dietary intake (3-days food records)	Participant/nutritionist		M	M^A,S^	M^A,S^	M^A,S^	M^A,S^	M^A,S^	M^A,S^	M^A,S^
Dietary intake (24 h dietary recalls)	Participant nutritionist			M^D^	M^D^	M^D^	M^D^	M^D^	M^D^	M^D^
Dietary intake (24 h weighing records)	Participant				I	I	I	I	I	I
Dietary intake (3-days food records)	Participant/nutritionist					I	I	I	I	I
Lactation education and support	Research team member		M	M	M	M	M	M	M	M
Nutritionist consultation	Research team member			M^D^	M^D^	M^D^	M^D^	M^D^	M^D^	M^D^
Food delivery	Research team member			M^D^	M^D^	M^D^	M^D^	M^D^	M^D^	M^D^
Antenatal breast milk expression video and Q&A session	Research team member		M^A^							
Infant feeding intention scale	Research team member		M							
Breastfeeding self-efficacy scale – short form	Research team member			M						
Edinburgh postnatal depression scale	Research team member		M	M	M	M		M		M
Perceived stress (PSS-14)	Research team member		M	M		M		M		M
Quality of Life Questionnaire (SF-36)	Research team member		M				M			M
Prenatal lactation medical history	Research team member		M							
Postnatal lactation medical history	Research team member			M	M	M	M	M	M	M

I, infant; M, mother; M^A^, mother in the antenatal breast milk expression (prenatal intervention); M^D^, mother in the dietary intervention (postnatal intervention); M^A,S^, mother in the antenatal breast milk expression (prenatal intervention) and mothers in the standard of care group (control group).

### 2.1 Study setting

This study will be conducted at the Arkansas Children’s Nutrition Center (ACNC), a USDA-ARS funded research center co-located on the Arkansas Children’s campus in Little Rock.

### 2.2 Eligibility and recruitment

Criteria for study inclusion are: women with BMI pre-pregnancy ≥25 kg/m^2^, <37 weeks of pregnancy with singleton fetus, age of 18 years or over, interested in breastfeeding, willing to follow study interventions and procedures, and residing in the Central Arkansas region.

Criteria for study exclusion are: pre-existing conditions (e.g., diabetes, hypertension, heart disease, thyroid disorders); use of recreational drugs, tobacco, nicotine, or alcohol; food allergies, food intolerances, or food preferences interfering with the meal plan; contraindication to breastfeeding (diagnoses, medications or supplements); history of breast surgery or radiation; congenital defects; preeclampsia or other hypertensive disorders of pregnancy.

Recruitment of participants will be conducted using IRB-approved tools at health care clinics, health care offices, health fairs, daycare centers, schools, universities, recreational centers, child retail stores, websites, or churches. Print or electronic advertisements may appear in newspapers, magazines, electronic media, social media and circulars. In addition, individuals who previously agreed to be contacted regarding future studies at ACNC or via the AR Research Registry at UAMS Translational Research Institute (TRI) will be contacted. All screening procedures will follow HIPAA compliant procedures.

The informed consent process will educate potential participants about the research study through IRB-approved recruitment methods and advertisements. Eligible potential participants will be contacted, and a copy (paper or electronic) of the informed consent will be provided to the potential participant prior to signing consent documents. A research team member will review all study procedures and the informed consent documents with the potential participant. Consent can be obtained in person or remotely (by phone or video conferencing with the consent form sent via email using the REDCap software). Potential participants will be given sufficient time to review the consent form, ask questions and receive clarification prior to signing any documents. No coercion or undue influence will be used during the consent process. If eligible and interested in enrollment, informed consent will be obtained and documented. During the consent process, participants will be given the option to allow for the use of their information and/or samples obtained from them or their child over the course of this study in future research studies related to breastfeeding, nutrition, weight status, growth, and development. The consent will be signed by both the participant and by the research team member obtaining consent. The participant will receive a copy of the signed consent form. A copy of the signed consent form will be stored in the participant’s research record. The randomization will occur at the first prenatal visit after consent. Randomization was generated using R statistical software (version 4.4.1) using a block of 6 to ensure equal enrollment of participants in each group for each season.

#### 2.2.1 Interventions

Standard of care group: Participants in this group will receive standard of care at their OB/GYN and pediatrician offices as well as standardized lactation support that include prenatal lactation education and postnatal lactation support provided by lactation specialist supervised by pediatricians who are board certified in Breastfeeding Medicine and Lactation.

Prenatal intervention - ABME: Participants in the prenatal group will receive standardized lactation support as described above. In addition, they will receive education and training (video and handout) about the ABME technique. At the prenatal visit, participants will watch a video describing ABME, meet with a lactation specialist to discuss the technique and practice the ABME technique during the research study visit. Participants will be asked to perform the ABME technique 2 times per day for 2–5 min. A redcap reminder will be sent daily to the participant to ask whether they were able to perform ABME and how much colostrum they expressed. Participants will be instructed to store the colostrum according to the human milk storage guidelines to use for their child after birth or to donate to the research team.

Postnatal intervention - dietary intervention: Participants in the postnatal group will receive standardized lactation support as described in the standard of care group. In addition, they will receive all foods needed to adhere to the study meal plan from 2 weeks to 6 months postpartum. The meal plan was designed to align with the DGA and consists of lean proteins, legumes, nuts, whole grains, fruits, vegetables, low-fat dairy, and healthy fats. All pre-packaged meals for lunch and dinner will be delivered weekly to the participant’s home. The meals will be supplemented with grocery items for breakfast and snacks items that the participant will pick up at the research center during their study visits. Participants will be encouraged to follow the provided meal plan 6 days per week. To support adherence and feasibility, participants will be allowed 1 day per week during which they may choose to consume foods that are not included in the meal plan. A nutritionist will meet with the participants to explain the dietary intervention at the enrollment visit (<36 weeks of gestation) and will meet with the participants at each research study visits to discuss barriers and challenges to following the dietary intervention. In addition, the nutritionist will contact the participants weekly during the first 4 weeks to address any concerns the participants may have. Compliance to the meal plan will be monitored by 24-h recalls (3 randomly chosen days around each research study visit).

All participants will receive standardized lactation support, including prenatal lactation education and postnatal lactation support, regardless of study group. The lactation support will reflect recommended practices and will allow standardization between women who would receive the support from their care team (doulas, lactation consultant) and those who wouldn’t due to access challenges or lack of awareness of the support availability, This study design will ensure our ability to assess the incremental benefit of the prenatal and postnatal interventions beyond the standard breastfeeding support, rather than to compare the intervention with no breastfeeding support.

### 2.3 Compensation and incentives

Participants will be compensated for their, travel and time spent at the research facility with one case of diapers after the prenatal visit and two cases of diapers for the postpartum visits up to 6 months. If participants are asked to return for a repeat visit, an additional one case of diapers will be provided. If participants are using cloth diapers, $25 will be provided on a reloadable, prepaid debit card each study visit. A bonus of $100 will be provided on a reloadable, prepaid debit card at the completion of the study if all visits are attended and all assessments are attempted. For participants experiencing transportation challenges, transportation to and from the center will be arranged using Uber Health.

### 2.4 Data collection, management, and analysis

#### 2.4.1 Data collection methods

Participants will attend up to 8 research study visits at the ACNC, one prenatal (≤37 weeks gestation) and 7 postnatal visits (0.5–6 months). After birth, moms will call to schedule the first postpartum visit, which includes the child’s first visit. Participants will also receive tele-lactation support at days 3 and 8 after birth in the form of a phone call from a lactation specialist. Questionnaires will be administered via online platforms such as REDCap for participant self-completion. Questionnaires administered by research staff member will be entered directly into the study database.

In cases such as early discontinuation of breastfeeding, the need to supplement with infant formula, or modification of the dietary intervention plan due to participant request, careful documentation as protocol deviations or participant-driven changes will be recorded. Breastfeeding-related challenges (e.g., pain/discomfort, engorgement, mastitis) will be assessed and addressed by a trained lactation specialist unless the participant is receiving lactation support elsewhere. Any challenges with adherence to the meal plan will be assessed and addressed by the nutritionist using motivational interviewing techniques. If these events result in changes of the intervention plan, they will be recorded. Any deviations will be documented, and sensitivity analyses will be performed to determine whether deviations significantly impacted the completeness, accuracy, and/or reliability of the study (52).

##### 2.4.1.1 Background information and release

Women who agree to participate will provide background information including their name, address, date of birth, race, ethnicity, education, income, child support, number of people living in their household, government assistance program eligibility, marital status, health history, household food and nutrition security (53) and quality of life (54). Mothers will also be asked to provide medical history, lactation history and medication history for themselves and the child at each postnatal study visit.

##### 2.4.1.2 Anthropometrics and vitals

All measures will be taken in duplicate or triplicate if not within acceptable agreement of measure. Maternal height will be measured to the nearest 0.1 cm with a wall-mounted stadiometer (acceptable agreement: ≤1.0 cm). Maternal weight will be measured to the nearest 0.1 kg using a standing digital scale that has been tared with clothing the participant is wearing (acceptable agreement: ≤0.1 kg). Maternal waist circumference will be obtained to the nearest 0.1 cm using a tape measure against bare skin or form-fitted shorts (acceptable agreement: ≤1.0 cm). Child length will be measured to the nearest 0.1 cm using a length board (acceptable agreement: ≤1.0 cm). Child weight will be measured to the nearest 0.01 kg using a tared digital scale (acceptable agreement: ≤0.01 kg). Child head circumference will be measured to the nearest 0.2 cm using a measuring tape headband (acceptable agreement: ≤0.2 cm).

Blood pressure and heart rate will be measured only in mothers with a digital blood pressure monitor (acceptable agreement: ≤6 mm Hg for systolic blood pressure or ≤4 mm Hg for diastolic blood pressure).

##### 2.4.1.3 Body composition

Maternal body composition will be measured using air displacement plethysmography (BodPod, Concord, CA) (55) which measures body weight and volume during a 2–5 min period. Child body composition will be measured using quantitative nuclear magnetic resonance (EchoMRI-AH, Echo Medical Systems, Houston, TX) (56), which measures body fat and lean mass and total body water during a 3-min scan using magnetic resonance technology with a magnetic field 1,000 times weaker than a clinical MRI.

##### 2.4.1.4 Biological samples

Colostrum and human milk samples: Colostrum collected prenatally may be donated by participants in the prenatal intervention group by bringing collected and stored samples at the first postnatal visit or any drop off visit. Alternatively, participants can use the stored colostrum to feed their infants after birth. Human milk samples will be collected by all participants at 2 weeks and 1 month postpartum. During these research study visits, participants will express a full breast, and a sample of approximately 10 mL of the expressed milk will be retained for research analyses while the remaining milk will be available to feed the infant. For all following study visits, beginning at 2 months postpartum, participants will be instructed to collect human milk samples over a 24-h period at home, prior to each study visit. Participant will express an entire breast at each feed for 24-h, alternating breast at each feed. From each of these expressions, approximately 6 mL of milk will be retained for research purposes, allowing enough human milk to be available to feed their child. After each expression, the expressed milk will be gently mixed by inverting the collection bottle and 3 mL will be transferred to a clear syringe and 3 mL transferred to an amber syringe. Detailed instructions will be provided to the participants to ensure standardized sample collection. Human milk samples collected from all feeding sessions during the 24-h period will be stored in the participant’s refrigerator (4 °C) and freezer (−20 °C), to adhere to the best practices for the assays that will be performed. Archived human milk samples will be analyzed for human milk macronutrients, micronutrients, and bioactives, and may be used for future analyses involving microbiome, metabolome, lipidome, transcriptome, and proteome.

Toenail: Standardized procedures will be followed for the collection of maternal nails (toenail) samples at 3 and 6 months post-partum. Prior to collection, clippers will be disinfected with a non-ethanol-based alcohol pad, and participants will be instructed to wash their feet. Participants will trim their nails as close to the nail bed as comfortable over a clean sheet of paper, and the clippings will be transferred into a foil tray. Approximately 100–150 mg of material (equivalent to ∼2 mm from all 10 nails) will be collected. Samples will be sealed in pre-labeled envelopes and returned at the next visit. Toenail samples will be analyzed for cortisol levels to assess chronic stress between the groups.

Urine and stool samples: Participants will be instructed to use standardized procedures for collection of urine and stool samples. Urine samples will be collected at the research study visits using a urine cup for the mother and urine self-adhesive collection bag for the infant. Urine samples will be centrifuged, aliquoted, analyzed for osmolality and stored at −80 °C for further analyses. Archived urine samples will be used to evaluate differences in urinary hormones, metabolome, transcriptome, proteome, and other biomarkers between groups. Stool samples will be collected at home before the study visit in OMNIgeneGUT and OMNImetGUT tubes (DNA Genotek, Ontario, Canada), which contain media and beads to homogenize and stabilize the samples at the point-of-collection, ensuring that microbiome and metabolomics profiles accurately represent the *in vivo* state, reducing bias and variability. After sample collection, the participant will seal the tube and homogenize vigorously to distribute the sample uniformly in the stabilization solution. The samples will be stored at room temperature until the next study visit. Archived stool samples may be used to analyze the microbiome, metabolome, transcriptome, or proteome to identify differences between the groups.

Blood samples: Standardized procedures will be followed by a trained phlebotomist to obtain maternal blood samples, up to 35 mL, under overnight fasted conditions. At each postpartum study visit, up to 35 mL of blood will be collected from the mothers. At the first postpartum visit (0.5-month), two blood draws will be performed: one before and one after breastfeeding to assess lactation-related hormonal responses. During the entire study, we will collect up to 245 mL total from the adult participant. At the 6-months visit, up to 3 mL/kg will be collected from the child. All biological samples will be processed, aliquoted, and stored at −80 °C until further analyses. Archived blood samples will be used to evaluate the differences in metabolites, hormones, transcriptome, proteome, epigenome, genome and other biomarkers between groups.

##### 2.4.1.5 Depression and perceived stress

Maternal depression symptomology will be evaluated using the Edinburgh Postnatal Depression Scale (EPDS), which has been shown to have high reliability (57) and validity (58). Participants’ level of perceived stress will be evaluated through the administration of the Cohen’s Perceived Stress Scale, which is a validated 14-item questionnaire examining the perception of stressful life events and levels of experienced stress over the course of the 4 weeks prior to the completion of the survey (59). Participants with elevated scores on the EPDS will be provided with mental health resources and referred to their primary care provider. Depression scores and perceived stress scores will be evaluated for differences between groups.

##### 2.4.1.6 Dietary assessment

24-h weighing records will be used to estimate infant daily human milk intake. Participants will complete weighing records for 24 h if they are nursing their child by weighing the child before and after each feeding. If the participants are expressing human milk and using it to bottle feed their child, they will record the weight of the bottle before and after each feeding on the weighing record instead of the weight of the child. Participants will be provided with a scale to complete the weighing records. For maternal dietary intake, participants will complete 3-days food records to record all foods, beverages, medications, and supplements (24-h for each day), following standardized procedures and instructions provided by the research team. After complementary feeding begins, mothers will follow the same methodology to record dietary intake of the child. Women in the postnatal dietary intervention group will be asked to report their intake on 3 random days (24-h dietary recalls at each study visit) to assess intervention adherence. During the 5.5-months intervention period, these 3-days 24-h dietary recalls will be completed in place of (not in addition to) the 3-days food records. For these recalls, participants will receive a text message, email, or phone call reminding them to report in detail everything they consumed on the previous day. Dietary intake will be evaluated by a trained research assistant for completeness and analyzed with the Nutrition Data System for Research (NDSR, Nutrition Coordinating Center, University of Minnesota, MN). Data entry into this software will be randomly audited (10%) by a trained research assistant to ensure quality control. Differences in dietary intake, dietary quality and diversity will be reported between the groups.

##### 2.4.1.7 Breastfeeding questionnaires

Participants will complete 4 questionnaires to assess breastfeeding outcomes. The Infant Feeding Intention Scale will measure strength and duration of a mother’s intention to exclusively breastfeed her child (60), the Breastfeeding self-efficacy scale–Short Form (BSES-SF) will assess the mother’s confidence in her ability to breastfeed (61). A lactation history questionnaire will evaluate lactation history, current breastfeeding history, formula supplementation, pumping, complementary foods, and plans for pumping when returning to work. Breastfeeding outcomes will be evaluated between the groups.

#### 2.4.2 Data management

All investigators and research team members will complete and maintain appropriate CITI training. All data will be recorded in standardized case report forms either on paper or electronically in protected databases such as REDCap. The database will be housed on a shared drive that is backed up nightly off-site Monday through Friday. Access to the server is password protected, as is access to the study database. Data and study samples will be labeled with a unique identifier (e.g., RN-101). This identifier will not be associated with the participant’s personal information. If a participant requests to no longer have their data stored, their data will be removed from the database. If participants provide consent, the entire screening form, including contact information and responses related to health history and eligibility, will be securely stored in a protected database for potential consideration for future IRB-approved studies. The screening data will be retained and used exclusively to assess eligibility for future studies. Data and samples will only be accessible by investigators, research assistants, and institutional oversight offices (e.g., IRB).

#### 2.4.3 Statistical analyses

Prior to analyses, data will be checked for potential outliers, aberrant measurements, missingness, and need for potential multiple imputations to assess the impact of missing data on model estimates. According to the prior published literature (62–72), we will explore maternal age, maternal race, parity, gestational weight gain, gestational age, maternal education, delivery mode, birth weight, mastitis occurrence, infant sex, return to work, and family support as potential confounders. Sensitivity analyses will be explored to evaluate the effect of maternal medication intake on the outcomes of interest. The main analysis will be performed according to the intention-to-treat (ITT) principle. In the ITT analysis, all participants are analyzed according to their initially assigned study arm at baseline, regardless of adherence to the study protocol. ITT analysis will be followed by per-protocol analyses, which would exclude participants who did not adhere to the interventions (<70% compliance). Compliance for the postnatal intervention will be calculated as the HEI score of consumed meals divided by the HEI score of the prescribed meals multiplied by 100, as described previously in our pilot feasibility study (40). Generalized linear models will be fitted to the observed study data. These models can account for fixed effects of BMI and intervention combinations as well as the correlated repeated time measurements of the longitudinal study design. Generalized linear models are also flexible for study responses that are not normally distributed. These models also allow for considerations of covariates to adjust estimates if necessary. In scenarios involving multiple comparisons, a false discovery rate (FDR) adjusted *p*-value will be used to assess statistical significance. All analyses will be conducted using R software, with statistical significance evaluated at α = 0.05.

Analysis of metabolite features will be conducted using linear mixed models, with appropriate correction for multiple comparisons (e.g., FDR-adjusted *p*-values). Multivariate procedures will also be employed. Visual discrimination of experimental groups will be assessed with Principal Component Analysis (PCA) or Partial Least Squares-Discriminant Analysis (PLS-DA). Model validity of the latter will be estimated with data splitting techniques (e.g., cross-validation, permutation testing, etc.). Weight Gene Correlation Network Analysis (WGCNA) will be used to summarize correlated metabolite features into unique modules that can be correlated to study outcomes (73, 74). Canonical correlation analysis (CCA) or sparse Partial Least Squares Regression (sPLSR) will be used to integrate metabolomics data with other data matrices, including other high-dimensional datasets (75).

#### 2.4.4 Sample size

To adequately power the study, 180 pregnant women with excess weight will be randomized into three groups of 60. The sample size is based on a mixed model of repeated measures with a general correlation structure assuming a group allocation of 1:1:1 ratio, alpha of 0.05, 80% power, seven assessment points, a between-groups effect size of Cohen’s *d* = 0.6 at post-intervention, a compound symmetry correlation matrix (rho = 0.8) and attrition rates. This resulted in a sample size of 60 participants per group to test the primary outcomes. Effect size estimates for the primary outcomes were based on our pilot study demonstrating that a healthy dietary pattern provided to lactating women with obesity (*N* = 13) modified concentrations of human milk leptin and specific human milk bioactives (40) and a RCT of an ABME intervention in women with pre-existing or gestational diabetes (21). We will recruit 257 women (assuming ∼70% retention and breastfeeding continuation) to achieve complete data on 180 mother-child dyads over a 5-years period.

#### 2.4.5 Monitoring

A Data and Safety Monitoring Plan is in place for this study. Adverse events, enrollment numbers, lost to follow up counts, and withdrawals will be monitored by the Clinical Study Coordinator. A continuing review will also be completed annually. Any adverse events and deviations will be reported to the IRB and included in the continuing review in compliance with IRB policies. Any adverse effects or unanticipated problems will be reported to the study Principal investigator (PI), the IRB, and the study sponsor in accordance with IRB policies. At all times, the IRB will be promptly provided with the information from the PI as needed.

### 2.5 Ethical considerations

This study will be conducted in accordance with all applicable government regulations and University of Arkansas for Medical Sciences (UAMS) research policies and procedures. The protocol, consent, questionnaires, participants’ biospecimens collections instructions, videos and handouts were submitted and approved by the UAMS Institutional Review Board (UAMS IRB-298382). All study-related communication, including the initial informed consent process and ongoing interactions will be conducted in a language understandable to the participant. For participants with limited English proficiency, a bilingual research staff member will conduct the visit and all the procedures. All study materials will be translated into Spanish by a certified translator.

### 2.6 Dissemination of findings

Findings will be disseminated via peer-reviewed manuscripts and presentations at scientific conferences, at which time, protocol amendments will be discussed as necessary.

## 3 Discussion

This paper outlines the design and methodology of a randomized clinical trial evaluating the effects of prenatal ABME and a postnatal dietary intervention on breastfeeding outcomes, human milk composition, and maternal and infant health outcomes in women with overweight and obesity. The findings will contribute to scientific evidence supporting the safety and feasibility of ABME, particularly among populations with excess weight who are at higher risk of poor breastfeeding outcomes. The ABME technique in non-diabetic pregnant women with excess weight is considered an innovative approach, since most studies investigating the outcomes of ABME are described in pregnant women with diabetes (22). In addition, the dietary intervention meeting the Dietary Guidelines for Americans, during the postnatal period, in women with excess weight may offer an opportunity to attenuate the effects of excess adiposity on Human Milk composition (40).

This protocol aligns with these previous trials by ensuring that ABME is introduced during the final weeks of pregnancy (typically after 36 weeks of pregnancy), and that the participants will be educated to perform it under trained professionals’ guidance. It is important to highlight that this study will include participants without diabetes, which can extend the findings of safety to a different population compared to that described in previous studies. In addition, in a systematic review, Sobik et al. (20) (*N* = 10 studies), noted that the successful implementation of ABME is closely tied to the quality of education and support provided. Depending on the results, ABME integrated into antenatal care may serve to enhance lactation confidence and early milk availability after birth. This is important because improving these breastfeeding outcomes, particularly early initiation, has been associated with better infant health outcomes, such as reduced neonatal mortality, lower incidence of infectious diseases (e.g., diarrhea and pneumonia), optimal brain development, and reduced risk of childhood obesity (76). On the other hand, this same review reported that the existing evidence base remains limited. The authors identified major weaknesses, including methodological heterogeneity, inconsistent timing of initiation (generally 34–36 weeks), and a predominance of studies focused on pregnant women with diabetes, which limits the generalizability of findings to other populations of pregnant women, such as those with different disease conditions or without health complications. In a scoping review, Foudil-Bey et al. (23) also reported that the published studies about ABME and the maternal and infant outcomes vary widely in their hypotheses, objectives, target populations, interventions and outcomes of interest.

Evidence from the past few years highlights the profound impact of maternal nutrition on the epigenome which may influence metabolic health over several generations (77). Barrès and Zierath (78) reviewed how environmental factors, such as diet, can modulate DNA methylation and histone modifications, thereby shaping gene expression programs that persist beyond immediate exposure. The critical period of the first 1,000 days of life, extending from conception to the second year of infancy, is a well-established window of opportunity for optimal maternal and child health. It represents a unique period of developmental plasticity, during which early nutritional and lifestyle interventions can profoundly influence the long-term health of both mothers and children (79).

Maternal and infant nutrition provide an opportunity to positively impact both maternal and infant health, with some studies demonstrating that women consuming a healthier diet have improvements in maternal health, milk composition, and infant health. Tahir et al. (80), in a prospective cohort, followed 354 exclusively breastfeeding mother-infant dyads from pregnancy to 6 months of life and assessed the maternal diet quality using the Healthy Eating Index-2015 (HEI-2015) at one and 3 months postpartum. The authors observed that higher maternal HEI-2015 scores were associated with lower infant fat mass and body fat percentage at 6 months, which suggests that maternal diet quality during lactation may beneficially modulate infant body composition in early life, offering potential targets for obesity prevention.

The Italian MEDIDIET study enrolled 282 mothers during lactation (6 weeks postpartum) and aimed to investigate the association between maternal dietary patterns and human milk composition. Although it was an observational study between 2012 and 2014 and did not include a formal intervention period the dietary data reflected the habitual intake in the postpartum period and suggested that high adherence to the Mediterranean diet was associated with lower proportions of saturated fatty acids and higher concentrations of monounsaturated fats and omega-3 fatty acids, especially docosahexaenoic acid (DHA), eicosapentaenoic acid (EPA) and α-linoleic acid (ALA) in milk (81). A study conducted by Karbasi et al. (82) examined the relationship between adherence to the Mediterranean diet during lactation and the concentration of bioactive compounds in human milk. This study included lactating women with infants under 6 months of age, with dietary intake assessed using a Mediterranean diet adherence score. The authors observed that higher Mediterranean diet adherence was associated with higher antioxidant capacity in both human milk and infant urine. In addition, a pilot study involving 13 exclusively breastfeeding women with obesity (BMI ≥ 30 kg/m^2^) at 4–5 months postpartum investigated the impact of a 4-weeks healthy meal plan (compliant with the DGA and the Mediterranean Diet) on human milk composition. Participants demonstrated a significant improvement in dietary quality, reduced energy intake and beneficial alterations in human milk composition, even with a short-term intervention (50).

Maternal diet during lactation may represent a critical modulator of the infant gut microbiota, acting through both alterations of microbial species in human milk and the transfer of microbial metabolites. A review indicated that although only one observational study directly assessed this relationship, experimental studies and evidence from interventions in pregnancy and lactation strongly support the hypothesis that dietary modulation of the maternal gut microbiome or probiotic intake can influence the human milk microbiome, which can contribute to shaping the infant’s gut microbiome composition and function (83). Taylor et al. (84) found that a varied, nutrient-dense maternal diet is associated with favorable change in the microbiota diversity, such as increases in *Bifidobacterium* following probiotic yogurt intake, and corresponding positive changes in infant gut microbial taxa associated with high fruit and vegetable consumption. On the other hand, maternal intake of high-fat diets or artificially sweetened beverages was correlated with less beneficial microbial profiles, such as *Bacteroides*. Given the central role of early-life gut colonization in immune development and metabolic programming, this highlights yet another reason why maternal dietary interventions during lactation may enhance infant health through this mechanism.

Based on the studies above, we hypothesized that a diet meeting the Dietary Guidelines for Americans during the first 6 months postpartum, will have multiple benefits. A diet rich in anti-inflammatory components and micronutrients during lactation may not only enhance maternal recovery, but also promote neurodevelopmental and metabolic health in infants, reflecting the preventive and intergenerational potential of this type of diet (77, 79). Although it is evident that this dietary pattern not only enriches the human milk with nutrients and bioactive compounds, optimizing early infant growth and health, but also enhances maternal metabolic health, potentially improving breastfeeding outcomes, several limitations remain. Most existing studies are cross-sectional or with small sample sizes, and heterogeneity in methodology. This limits comparability and generalizability of the study findings. Further, there is a significant gap in the scientific studies examining long-term infant outcomes and adherence to the Mediterranean diet during lactation (85).

Therefore, by improving maternal dietary practices during the postpartum period with evidence-based principles for the first 1,000 days, this randomized clinical trial promises to contribute important data on the mother-infant-human milk triad. Future studies should explore how the ABME and the maternal healthy diet during lactation influence the bioactive profile of human milk, and in turn the long-term metabolic and neurodevelopmental outcomes. Finally, studies should also consider the practical challenges of dietary adherence in postpartum populations.

### 3.1 Strengths

This study has several strengths. It is a randomized controlled trial, with a longitudinal follow-up until 6 months postpartum for both mother and child. It will offer participants personalized interventions: with lactation support for all groups, ABME education and support for the prenatal group, and nutrition provision and support for the postnatal group. The use of advanced analytical techniques for bioactive milk components profiles and body composition assessments (BodPod and MRI) provides robust biological measures.

### 3.2 Contingencies

Certain limitations should be considered. The ABME technique is not a practice widespread between pregnant women, and many pregnant women may not feel comfortable expressing colostrum. In addition, participants may not be willing to follow a dietary meal plan for almost 6 months. Adherence to the ABME and dietary intervention will be monitored to consider in the interpretation of the outcomes and results. Furthermore, sociocultural and environmental factors, such as maternity leave and return to work, family support, and access to breastfeeding support services, influence breastfeeding initiation, exclusively and continuation (86–89). Although these factors are not the primary focus of this study, they will be considered when interpreting the results.

## 4 Conclusion

This randomized controlled trial aims to assess the effects of ABME and postpartum dietary interventions on breastfeeding outcomes, human milk composition, infant growth in women with excess weight. These findings will contribute to addressing key gaps identified by the Dietary Guidelines Advisory Committee for pregnant women with excess weight and highlight the importance of personalized strategies to support lactation and early-life nutrition. The findings from this trial will contribute to improving maternal and child health trajectories, particularly in populations at increased risk of breastfeeding challenges.
